# Ladies First: Coerced Mating in a Fiddler Crab

**DOI:** 10.1371/journal.pone.0155707

**Published:** 2016-06-15

**Authors:** Christina J. Painting, William Splinter, Sophia Callander, Tim Maricic, Marianne Peso, Patricia R. Y. Backwell

**Affiliations:** 1 Research School of Biology, The Australian National University, Canberra, ACT, Australia; 2 School of Biological Sciences, University of Auckland, Private Bag 92019, Auckland Mail Centre, Auckland, New Zealand; 3 Department of Biological Sciences, Macquarie University, North Ryde, NSW, Australia; University of Missouri, UNITED STATES

## Abstract

In some species males increase their reproductive success by forcing females to copulate with them, usually by grasping the female or pinning her to the ground to prevent her from escaping. Here we report an example of males coercing copulation by trapping a female in a confined space. During mate-searching, female *Uca mjoebergi* fiddler crabs visit males and choose whether or not to enter their burrow for inspection. Males typically enter the burrow first and we found that 71% of females will follow him down and 54% decide to stay and mate. However, some males use an alternative tactic where he will wait for the female to enter the burrow first, after which he traps her inside. Although a significantly lower percentage of females will enter a burrow following this behaviour (41%), upon entry 79% females that enter will become trapped and almost all of these females (90%) produce a clutch of eggs. Our observations suggest that males are able to gain fertilisations from females that may not have remained in the burrow by trapping them and coercing them to mate.

## Introduction

The main limitation on male reproductive success is access to females. This selection pressure has led to an incredible diversity of behaviours, weapons and ornaments that assist males in winning the competition for females. We widely recognise competition for mates in two forms: (i) intrasexual male-male competition for resources or direct access to females, and (ii) intersexual courtship displays in which males compete for female attention [1]. During courtship interactions a female can compare males and select her preferred partner. However, males can employ a third tactic to gain access to females: sexual coercion in the form of forced copulation [2]. Sexual coercion allows males that might not otherwise have been chosen as a mate to increase their reproductive success. This type of mating system has been observed across many animal taxa including insects [3–6], reptiles [7], fish [8], birds [9, 10], and mammals [11].

During forced copulations a male can use his body weight to press the female to the ground until she submits [7, 9], or he can use modified body parts such as abdominal pinching organs ("gin traps"; [5]) or clasping structures [12] which aid him in holding onto the female to prevent her escape. Forced copulations are more common when a female’s movement is restricted, such as when animals are housed in captivity [13].

The mating system of some fiddler crabs (genus *Uca*) and other ocypodoid crabs, where males dig burrows and attempt to attract wandering females to enter their burrow prior to underground mating, may have allowed males to take advantage of females entering a confined space to coercively mate. In some species female crabs will enter a male’s burrow first, after which he follows her down and mates with her underground [14–18]. Forced copulation *per se* is unlikely in crabs because females must open their abdomen to expose their vulvae for copulation to occur [19, 20]. However, males may effectively coerce mating by trapping females, restricting their movement and preventing their escape. Other forms of coercive mating occur among ocypodoid crabs, including startling, herding, and capture ([21–25], but see [26]). For example, some male sand bubbler crabs (*Scopimera globosa*) wander the sand-mud flats where they capture females, carry them back to their burrows, and force them underground to mate [22]. Here we explore the use of trapping as a form of coercive mating in the banana fiddler crab, *Uca mjoebergi*.

Among male *U*. *mjoebergi* there are high levels of competition to gain the attention of females, which wander through a population visiting up to 20 courting males before selecting a mate [27]. During these walks a female encounters an abundance of males all vying for her attention by moving towards her and waving their large, major claws in an attempt to coax her back towards their burrows for inspection. A female’s decision to approach a male is based on multiple male traits including body size, claw colouration, wave rate and wave leadership [27–31]. After approach, a female may enter a male’s burrow and, either decide to stay in the burrow to mate, or leave to continue searching. This decision is based on burrow features such as volume, sediment density and structural complexity [27, 32]. Since females remain in the male’s burrow to incubate their eggs after mating, it is important that she selects a burrow with suitable conditions for the development of her embryos [33, 34].

Male *U*. *mjoebergi* respond to approaching females by either (i) entering his burrow once she nears the entrance and waiting for her to follow him down (‘enter first’); or (ii) stepping aside at the burrow entrance and waiting for the female to enter first while continuously waving his claw, and then following her down the burrow shaft (‘step aside’). Our preliminary observations suggested that females were less likely to enter a burrow when the male stepped aside, which was in line with a previous study on another fiddler crab, *U*. *lactea perplexa*, which also displays this mating system [15]. We therefore posed the question: why would a male decrease the chance that a female enters his burrow for inspection given the importance of this behaviour as a prerequisite to mating? We hypothesized that for this behaviour to have evolved and be maintained in the population, there must be a fitness benefit to the males that perform it. A male’s burrow shaft is narrow and it is possible that he can trap a female in his burrow if he enters after her. We therefore predicted that the decreased likelihood of the female entering the burrow before the male would be compensated for by the increased chance of forcing the female to mate by trapping her underground. Here we examine the likelihood of the female entering a burrow and remaining to mate when the male steps aside or enters the burrow first. We also determined whether male body size related to the type of mating tactic used because we hypothesized that a specific subset of males perform the alternative ‘step aside’ tactic. We predicted that these males would be significantly smaller in body size (and therefore have smaller, less attractive claws [35]) than males entering the burrow first, to allow them to have mating opportunities that they may not have otherwise had [2, 3]. In relation to this we also predicted that males that step aside would perform this behaviour consistently over time. Similarly, we determined whether female body size influenced the male’s decision to enter first or step aside, and whether this related to the probability that a female entered the burrow and remained to mate. Finally, to determine whether females actually copulated with males when trapped underground (rather than just remaining there until they could escape), we inferred fertilisation success by checking for the production of an egg clutch for a subset of females that were trapped or entered burrows by choice.

## Materials and Methods

### Study site

We studied a population of *U*. *mjoebergi*, at East Point Reserve, Darwin, Australia (12.24°N, 130.49°E) from September to December 2012 and from September to October 2014. Our research was done with permission from the Darwin City Council (Permit No. 2322876).

### Behavioural observations

To determine whether male mating tactic affected the probability of females entering a male burrow and remaining to mate, we first identified mate-searching females (wandering females that had approached at least one male) and followed them as they visited another displaying male, staying at least 1.5 metres away to avoid disturbing the crabs. We recorded the behaviour of the female and the male for each visit. For the females, we recorded whether or not the female completely entered the male’s burrow shaft after approaching, and then whether the female stayed to mate after entering the burrow. We also recorded whether the male descended in the burrow as the female approached the burrow entrance (enter first), or whether the male stepped aside as the female approached and waited for her to enter the burrow first (step aside). We considered all occurrences where a female stayed underground for more than 10 minutes, the male displayed guarding behaviour, or sealed the pair underground as an indication that mating had occurred. Previous work has shown that these behaviours are reliable indicators of mating [27]. After a single observation for each female (N = 144), where possible we captured and measured the female (carapace width to the nearest 0.1mm) by blocking her burrow entrance when she was on the mud surface after she left the male’s burrow, or by digging her up in the burrow if she stayed down to mate, for a subset of the burrows (see below). If the burrow entrance was damaged, we repaired it before returning the crab to the burrow.

### Determining mating success

To compare mating success between the two male mating tactics we determined whether females extruded eggs for a subset of the observations described above. For 10 of the sequences in which the male stepped aside and 18 sequences during which the male entered the burrow first, we marked burrows with wooden skewers and a plastic enclosure after the male sealed himself and the female underground. We waited several days for the male to leave the burrow before digging up the female to check if she had extruded a clutch of eggs, which are visible on the underside of the crab. Unfortunately, the burrows were destroyed in the process of capturing the female. We therefore made a new burrow for each female using a 1cm diameter rod pushed into the sediment at an angle of 45° for 15cm (the ‘normal’ burrow conditions for this species). Previous observations have confirmed that these females stay and incubate in these created burrows with no adverse effects (P. R. Y. Backwell personal observations).

### Determining consistency of male mating tactic

To determine whether males switch tactics between stepping aside and entering first, we marked 124 male burrows using small coloured flags and observed them as they displayed to wandering females. For each female visit, we noted whether the male entered first or stepped aside. We continued watching the male until he received a second visit from a different female and again noted if he entered first or stepped aside. We obtained data on 37 males that received repeated visits.

Finally, to determine whether male body size affected behavioural tactics, we also observed and recorded the behaviour of 24 males in response to mate searching females (N = 16 females), after which we captured the pair and measured their carapace widths and the male’s major claw length.

### Data analysis

Binary generalised linear models (GLMs) with a logit link were used to determine the probability that (1) a female enters or does not enter a male’s burrow in relation to his mating tactic (male in first vs. step aside), and (2) a female stays to mate or leaves the burrow in relation to the male’s mating tactic. Female body size and the interaction between female size and male mating tactic were also included in both models as fixed effects. To determine whether male body size affected his decision to step aside or enter the burrow first, we used a binary generalised linear mixed model (GLMM) with logit link in the package *lme4* [36] to account for non-independence in the dataset due to repeated measures of female visits (N = 24 males, N = 16 females). Carapace width only was used a measure of male size in this analysis due to very high collinearity between carapace width and major claw length (r = 0.98).

We used likelihood ratio tests to determine the influence of each fixed effect on the fit of the model using the package *lmtest* [37]. The final model presented includes only the variables that were found to significantly affect the response variable in question. Effect sizes are reported as odds ratios with 95% confidence intervals (CI; [38]). Means presented are ± standard error of the mean. All analyses were performed in R 3.1.3 [39].

## Results

**Fig 1:**
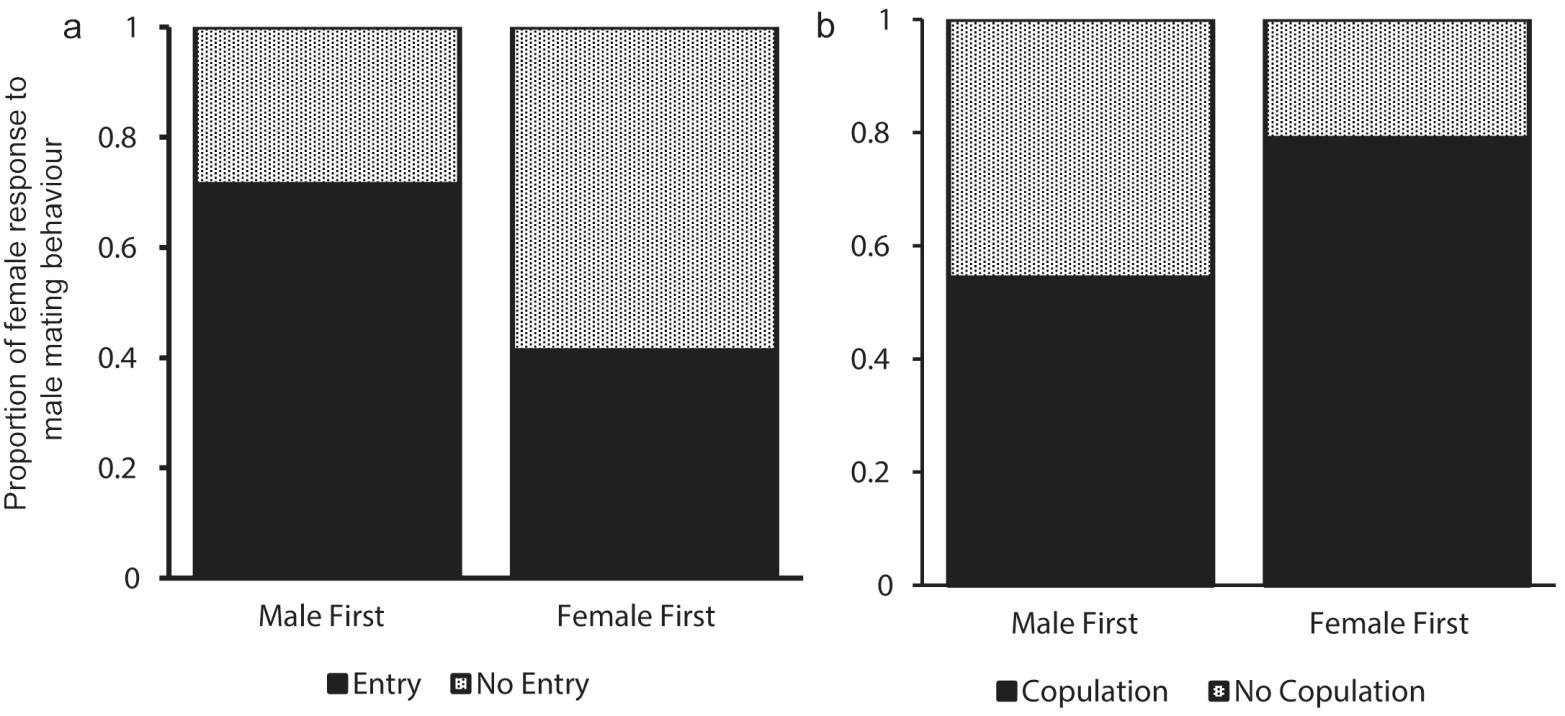
Response of female *Uca mjoebergi* to male mating behaviour. (*a*) Proportion of females that entered or did not enter the male burrow, (*b*) proportion of females that remained in burrow to presumably copulate or left before copulating.

Males entered the burrow first (98/144, 68%) more frequently than stepping aside and waiting for the female to enter first (46/144, 32%). Females were significantly more likely to enter a male’s burrow when he entered it first (71% females entered) than when he stepped aside (41% females entered; Table 1; Fig 1a). The size of the female did not relate to her decision to enter the burrow (or not) in response to the male’s behaviour (χ ^2^ = 2.39, *P* = 0.12), and there was no significant interaction between female body size and male mating tactic (χ ^2^ = 0.01, *P* = 0.93).

**Table 1:** Results of binary GLMs determining the probability of female *Uca mjoebergi* entering the male’s burrow and remaining to mate in relation to male mating tactic.

	Estimate	SE	z	*P*	Odds Ratio (±95% CI)
Probability of female entering burrow (n = 144)					
Intercept	0.92	0.22	4.10	<0.001	
Male behaviour	-1.27	0.37	-3.39	<0.001	3.55 (1.72–7.49)
Probability of female remaining to mate (n = 89)					
Intercept	0.17	0.24	0.72	0.47	
Male behaviour	1.15	0.61	1.88	0.06	3.16 (1.03–11.94)

Females were less likely to stay and mate with a male when he entered the burrow first (54% stayed to mate) than when he stepped aside (79% females stayed to mate; Table 1; Fig 1b). Although the *P* value was marginally above 0.05, females that entered when a male stepped aside were approximately three times more likely to stay and mate than if males entered the burrow first (Table 1). Female body size did not significantly affect the probability of her staying to mate once in the burrow (χ ^2^ = 0.51, *P* = 0.48), and there was no significant interaction between female body size and male mating tactic (χ ^2^ = 2.66, *P* = 0.10). A male’s decision to step aside or enter the burrow first was not significantly related to his carapace width (male in first: mean = 10.67 mm ± 0.33, N = 14; male steps aside: mean = 10.26 mm ± 0.50, N = 10; χ ^2^ = 0.65, *P* = 0.42).

There was no difference in the probability of females extruding eggs after staying in a burrow with a male that stepped aside (9/10 females were ovigerous), compared to those who mated with males that entered the burrow first (17/18 females ovigerous, Fisher’s exact test: *P* = 1.0, odds ratio ± 95% CI = 0.54 [0.006–46.13]).

Of 37 males visited by two different females, 35 males used the same tactic on both occasions (N = 28 entered first on both visits; N = 7 stepped aside on both visits), while two males switched tactics from step-aside to entering first between the two visits. This is significantly less switching than expected by chance (Chi-square test, χ ^2^ = 22.03, *df* = 1, *P* < 0.001, φ = 0.85).

## Discussion

We found that the mating tactic performed by a male crab as a female approaches his burrow has a significant effect on the probability that the female will enter the burrow and a marginally significant effect on whether she will remain in the burrow to mate. Females were more than 3 times less likely to enter a male’s burrow when he stepped aside and waited for her to enter first, compared to occasions when a male entered the burrow first. Conversely, when females did enter a burrow after males stepped aside, they were more than three times likely to remain in the burrow and mate. These results strongly suggest that entering a male’s burrow first reduces the probability that a female will leave the burrow after sampling it since females are effectively trapped underground in the narrow burrow shaft when the male follows her in. Several types of coercive mating behaviour have been observed in fiddler crabs, and other ocypodoid crabs, such as herding, carrying a female to the burrow, and startling [21–26]. Males stepping aside and waiting for females to enter the burrow has also been observed in several other ocypodoid crab species [14–18], and our results are in line with a study on *U*. *lactea perplexa* that similarly found that mating was more likely to occur when females entered the burrow first, probably due to an inability of females to escape [15].

Aggressive tactics such as coercion can be under selection when these tactics allow those males to mate that would otherwise likely have been avoided by females [2]. Body size has been shown to be related to the probability of a male employing sexual coercion when this is just one of several tactics that can be used to secure mates. For example, forced copulation in camel crickets (*Pristoceuthophilus marmoratus*) is recognised as a condition-dependent alternative mating tactic because only small males perform this behaviour, despite it being logical to assume larger males would be better able to physically restrain females [3]. Contrary to our predictions, we did not find a relationship between male body size and mating tactic in *U*. *mjoebergi*, similar to two previous studies on related fiddler crab species [14, 15]. Female fiddler crabs have already exhibited a certain level of choice by approaching a male’s burrow as he waves her in. However, females reject a large proportion of males after entering and assessing burrow quality [27, 32]. Therefore, one possibility for the evolution of step aside behaviour is that males with burrows of poor quality perform these tactics as a way of securing mates, because females would be less likely to stay and mate after initial burrow entry and inspection. Although females are less likely to enter a male’s burrow if he steps aside, 41% of the females we observed did enter, suggesting that this tactic may not be that costly for males because at this point they have the opportunity to trap females inside the burrow and secure a mating opportunity.

An important question that arises from these observations is why females risk entering the burrow at all if there is a likelihood of being trapped with an unsuitable mate? Forced copulation can lower female fitness [40] and not surprisingly these increased costs drive arms races between males and females [2]. It therefore seems logical to assume that female *U*. *mjoebergi* would avoid situations where they could be trapped and forced to copulate. In some species, males force young, inexperienced females into copulation. For example, in *Drosophila melanogaster* fruit flies males force teneral females to copulate, whereas older, sexually mature females appear to choose their mates [4]. Although we did not measure age directly, body size is often used as a proxy for age in crabs [41]. In line with two previous studies [14, 15], female body size was not related to the probability that male *U*. *mjoebergi* would perform step aside behaviour, suggesting that it is not small or young females being coerced more often by males. An alternative (although unlikely) reason for a female’s apparent willingness to enter burrows before a male in some situations is that when trapped underground they are able to tests a male’s strength and vigor as they attempt to exit the burrow. It is important to consider that females may benefit from apparent male coercion, especially in the absence of obvious female resistance. For example, herding and capture by male ocypodoid crabs, where they intercept wandering females and guide or carry them to their burrow to mate, has been interpreted as coercion because females were observed struggling to escape [19, 24]. However, it is possible that females benefit by being able to test a male’s strength during resistance [21, 23], or that herding behavior is actually a form of courtship guiding rather than coercion [26]. Distinguishing between coercion and female choice can be extremely difficult and studies that have attempted this found little evidence of female choice [6, 8, 42, 43]. However, Eberhard [12] argues that the inability of most males (at least in insects) to force females to open their genitalia for intromission suggests that females may be using resistance to screen suitable mates rather than resisting to avoid copulation altogether.

Mating underground in complex burrow systems makes direct observation of coercive mating exceptionally difficult. Despite being trapped underground, it is possible that females could prevent copulation by refusing to open her abdominal flap to allow the male access to her gonopores [19]. However, we think this is unlikely as nine of the 10 step-aside matings that we monitored resulted in a clutch of eggs being produced. This is a comparable rate to that seen in the overall population both from our current study (17/18 females (94%) produced a clutch of eggs after remaining in the male’s burrow) and from a previous study (16/20 females (80%) produced eggs after staying in burrow [44]). It therefore appears that females who are trapped inside the burrow by the male are prevented from leaving until they relent and allow the male to copulate. In this scenario, the female is likely coerced to mate with a male she would probably have rejected. We do, however, acknowledge that although females will extrude eggs after mating with males that have stepped aside, we do not know if there are any costs to a coerced female in terms of reduced fecundity or survival. This could be especially apparent if females are forced to mate with males providing a poorer quality burrow, given how important this is for embryo development [27, 33, 34].

A possible future initiative to indirectly test the hypothesis that step aside matings are coercive would be to measure the average time that the male is in the burrow with the female in step aside versus male first pairings (John Christy, personal communication). Males usually remain with females until they extrude eggs to prevent intruder males from entering the burrow to mate, therefore ensuring paternity [45]. Therefore, if step aside matings are coercive we would expect that males spend longer in the burrow with females while they presumably resist mating and try to escape, than compared to male first matings. Nakasone and Murai [15] measured the duration of step aside and male first pairings combined and found that almost 86% of pairings lasted two days or less (minimum 1 day, maximum 4 days). It would therefore be difficult to detect an effect of female resistance time using this method given there is little variation around pairing times.

Males seldom changed their mating tactic (<7%). The majority of males (66%) consistently entered the burrow before the female; with fewer males consistently stepping aside (27%). Similarly, Murai et al. [14] found that *U*. *tetragonon* fiddler crabs would sometimes change between enter first and step aside tactics, but like *U*. *mjoebergi*, most males repeatedly used the same behaviour. However, both studies only monitored males within a single day and it is possible that males switch tactics between days, throughout the tide cycle or over a male’s lifetime. As previously discussed it is possible that males choose mating tactics based on the quality of their burrow or on environmental conditions. Therefore, studies tracking males over longer periods of time are required to determine how the choice of tactic relates to the dynamic way in which males compete for the best burrows, as well as environmental conditions such as timing within a tide cycle.
